# Alexis St. Martin

**Published:** 1881-08

**Authors:** 


### EDITORIAL DEPARTMENT.

"If thou hast truth to utter, speak;
And leave the rest to God."

Alexis St. Martin.—The death of this world-renowned individual is announced in the medical exchanges and will be a source of regret to many. No man, probably, ever lived whose life conferred more real and permanent advantages upon the physiology of the human digestive process than that of Alexis St. Martin. A Canadian by birth, accident made him a benefactor of his race and caused his name to be a household word throughout Christendom. For more than half a century the cicatrised parieties of the abdomen, and the opening inflicted through the integuments and into the stomach, remaining open, opportunity was offered for seeing the stomach perform the wondrous process of digestion. In this way many doubts, ambiguities, and absurd ideas were dispelled, and facts learned in regard to the subtle chemistry of digestion, of which no knowledge, and in some instances, at least not even conjecture, had existed, in regard to the mysterious function. Thus the time necessary for the digestion of various articles of food was easily learned, and the effect of certain irritants, notably alcohol, upon the inner coating of the stomach, readily seen and understood. Such a benefactor of his fellows merits something more at their hands than a mere record of the great and lasting good he has conferred! However unwillingly or unintentionally he became the instrument of science, his accident—or shall we not call it the Providence—made revelations from Nature's arcanum which were never known before in the history of mankind. This humble and uncultured Canadian merits, and should receive, greater distinction at the hands of his fellowmen, than he deserves who carves through human heads and human hearts his path to glory.

